# Dual host and pathogen targeting by MEK1/2 inhibitors

**DOI:** 10.1128/iai.00064-26

**Published:** 2026-06-04

**Authors:** Eryn Zuiker, Matthew E. Long

**Affiliations:** 1 Department of Microbial Infection and Immunity, The Ohio State University Wexner Medical Center 12306, Columbus, Ohio, USA; 2 Dorothy M. Davis Heart and Lung Research Institute, The Ohio State University Wexner Medical Center 12306, Columbus, Ohio, USA; University of California Merced, Merced, California, USA

**Keywords:** MEK1/2, macrophage, *Staphylococcus aureus*, neutrophil, antimicrobial agents, antiviral, anti-inflammatory

## Abstract

Therapeutic strategies that manipulate the host response to infection to reduce inflammation and host driven tissue damage may reduce disease pathogenesis. However, therapeutic approaches that have dual host-modulatory and antimicrobial therapeutic effects remain limited. Mammalian targeting MEK1/2 inhibitor compounds represent one class of small molecules that have now been recognized to have anti-viral effects through disruption of viral replication in epithelial cells and have direct anti-bacterial effects on gram-positive bacteria. MEK1/2 inhibitors elicit anti-inflammatory effects during *in vivo* infection and injury models that also include models of infection with gram-negative bacteria and parasites. This review aims to summarize progress made in understanding the effects of MEK1/2 inhibitor compounds on host innate immune cells and will highlight experimental models of infection or injury in which MEK1/2 inhibitors have been evaluated. The direct and indirect antimicrobial effects of MEK1/2 inhibitor compounds and the emerging recognition of different roles of MEK1 and MEK2 in programming cellular responses will be identified and discussed.

## INTRODUCTION

Poor outcomes following many infections can result from detrimental inflammatory responses that contribute to tissue damage without clearing the infection. The global rise in antimicrobial resistance in bacterial species remains an ongoing problem with limited development or approval of new antimicrobial compounds. Acute viral infections also pose significant health challenges that have few therapeutic options. Combined, there are limited clinical options for pathogen-targeting therapies. Alternative therapeutic approaches that target shared features of microbial infections could reduce the burden of disease; one example of a host targeting therapy is an IL-6 receptor antagonist, which underwent large clinical trials for the treatment of COVID-19 (1). However, therapeutic compounds that exert both microbial-targeting and host-targeting effects may allow for additional therapeutic benefits. The mammalian targeting MEK1/2 inhibitor compounds are small molecules that may be therapeutically repurposed for dual host and pathogen targeting effects.

## PHARMACEUTICAL TARGETING OF THE MAMMALIAN MEK1/2 PROTEINS

MEK1 and MEK2 are mammalian serine/threonine protein kinases that have pleiotropic functions in multiple cellular responses (2), and eukaryotic conservation of MEK1 and MEK2 has been well described (3). MEK1 and MEK2 are activated and form heterodimers and function as key signaling mediators in the RAS-RAF-MEK-ERK signal pathway (4–6). As mammalian protein kinases gained recognition as critical regulators of many key cellular signaling pathways altered in human diseases, and especially cancer, small molecule inhibitor compounds targeting these proteins, including MEK1/2, have been widely developed by the pharmaceutical industry (7). The primary focus for the development and translational use of MEK1/2 inhibitor compounds has been on diseases broadly categorized as RASopothies (8). RASopothies include diseases caused by both germline mutations, which typically result in developmental abnormalities, and somatic mutations, which are commonly found in oncogenesis, previously reviewed in more detail (8). Developmental defects due to RASopothies may be therapeutically challenging to treat due to their manifestations during early development. In contrast, the somatic mutations associated with cancer often result in RAS pathway overactivation and have a prolonged therapeutic window for treatment. MEK1 and MEK2 are central mediators of RAS pathway activation and, therefore, have been key targets for the development of pharmacologic inhibitor compounds (9). As a result of these research efforts, small molecule MEK1/2 inhibitor compounds targeting an allosteric pocket specific in MEK1 and MEK2 that differs from the ATP-binding pocket commonly found in many protein kinases have allowed for increased specificity of inhibitors targeting MEK1/2 while reducing off target effects (7).

There are currently six unique FDA-approved MEK1/2 inhibitors (7, 9). Trametinib (GSK1120212) was approved in 2013 as a treatment for melanoma or glioma with *BRAF* mutations and was followed by approval of Cobimetinib (CDC-0973) (10) and Binimetinib (MEK162) (11). Selumetinib (AZD6224) was approved in 2020 for the treatment of type I neurofibromatosis (12). Mirdametinib (PD0325901) was also approved for type I neurofibromatosis treatment in both pediatric and adults in 2025 (7, 13). Avutometinib inhibits the function of MEK1/2 and RAF and received accelerated approval for use in 2025 for treatment of adults with recurrent low-grade serous ovarian cancer when provided in combination with Defactinib, a FAK, and Pyk2 inhibitor (9, 14). In addition to these six compounds, numerous other MEK1/2 inhibitor compounds have been developed over the last several decades, and clinical studies have evaluated MEK1/2 inhibitors for arthritis treatments (15). At least one MEK1/2 inhibitor compound, called ATR-002, has been used in a human clinical trial to limit severe pneumonia due to viral infection (16). Overall, while the clinical use of MEK1/2 inhibitor compounds is encouraging, the effects of MEK1/2 inhibitor compounds on immune cells in these translational contexts are not fully understood, and additional investigations into the potential benefits or consequences of MEK1/2 inhibitors should continue to be explored. The *in vivo* application of MEK1/2 inhibitor compounds in experimental models of disease are summarized in Table 1 and highlight both the broad scope for which these compounds have been studied and variability in the specific compound tested and delivery methods. While this variability can result in challenges comparing results from different studies, overall beneficial host-targeting and pathogen targeting properties of MEK1/2 inhibitors have emerged over the last several decades of research.

**TABLE 1:** Experimental *in vivo* models with MEK1/2 inhibitor treatments

Animal model	Pathogen or injury model (location)	Methods and inhibitor delivery	Results	Citation
**U0126**				
Male BALB/c mice	Yellow Fever Virus (intracranial)	Pre-treated with U0126 1 day prior to infection, and daily treatment (via tail vein)	↓ Viral Titers in brain tissue	(17)
Young Male and Female Swiss Albino mice	Rabies virus (intracerebral)	U0126 on Days 0, 2, 4, and 6 post-infection	Less Severe clinical signs ↑ Survival ↓ Viral Titers ↓ CXCL10, TNFα, iNOS	(18)
Female C57BL/6 mice	Influenza A virus (intranasal)	Pre-treated with U0126 1 h before infection (via inhalation chamber)	↓ Virus Titer in the lung 24 h p.i. ↑ Survival	(19)
C57BL/6 mice	Influenza A virus (intratracheal)	Pre-treated with U0126 1 day prior to infection, on day of infection, and then daily	↓ Viral Titer in BAL ↓ TNFα	(20)
Female BALB/c mice	*L. amazonensis* (hind footpad)	U0126 i.p. daily for 7 days		(21)
		Treatment start on 2nd or 3rd week of infection (follow to 6 weeks)	↑ Lesion Size ↑ Parasite Burden Worse histological skin scores	
		Treatment start on 4th week of infection (follow to 5 weeks)	↑ Lesion size ↑ Parasite Burden Worse histological skin scores ↑ IFN-γ	
Female BALB/c mice	*L. amazonensis* (hind footpad)	U0126 i.p. weekly for 5 weeks starting 18 days post-infection	↓ Lesion Size ↓ Parasite Burden	(22)
		U0126 i.p. weekly for up to 6 weeks	↓ IL-10 mRNA (lesions, feet)	
Female J_H_ mice (BALB/c background)		U0126 i.p. every 7 days from day 20–48 post-infection with 200 µL anti-LA serum	↓ Antibody-induced lesion swelling ↓ Antibody-induced increase in parasite burden	
BALB/c mice	*E. coli* LPS-ALI (aerosolized)	Pre-treated with U0126 or PD98059 i.p. 2 h prior to LPS challenge	U0126: ↓ Total BAL cells, neutrophils, macrophages, and lymphocytes ↓ MPO Activity in lung tissue ↓ Albumin levels in BALF	(23)
			PD98059: ↓ Total cells in BALF, neutrophils ↓ MPO Activity in lung tissue	
**PD98059**				
Male CD mice	Zymosan (i.p.)	Treated with PD98059 i.p. 1 h and 6 h post-zymosan Harvest: 18 h for non-septic shock analysis 12 days for toxicity and mortality analysis	↓ Exudate (volume) ↓ Total peritoneal exudates cells ↓ IκB-α, NF-κB p65 (ileum, lung) ↓ NO, iNOS (lung) ↓ TNFα, IL-1β (plasma) ↓ Development of systemic toxicity ↓ Mortality	(24)
Male CD mice	Bleomycin (intratracheal)	Treated with PD98059 by i.p. 1 h post-bleomycin, and daily for 7 days	↓ Pulmonary lesion severity ↓ Edema ↓ Neutrophil infiltration ↓ IL-1β, TNFα, NF-κB p65 ↓ iNOS expression ↓ Body weight loss	(25)
		Treated with U0126 i.p. 1 h post-bleomycin, and treated daily for 7 days	↓ Lung tissue damage	
Adult Male CD1 mice	Carrageenan-induced lung injury	Treated with PD98059 i.p. 1 h post-injury	↓ Lung injury (edema, PMN infiltration, TNFα, IL-1β) ↓ iNOS expression ↓ Lipid Peroxidation	(26)
Male Sprague Dawley Rats	Cecal ligation and puncture (CLP)	Pre-treatment with PD98059 i.p. 30 min prior to CLP	↓ Lung injury score ↓ MPO activity	(27)
Sprague Dawley Rats	*H. pylori* LPS (intragastric)	Pre-treat with PD98059 intragastrically 1 h prior to challenge and twice daily post-challenge for 4 days	↓ Gastric inflammation (dose-dependent)	(28)
C57BL/6 mice	*P. berghei* ANKA (PbA) Experimental cerebral malaria (ECM) model	Treated with PD98059 i.p. starting at 6 h post-infection	PD98059: Negligible disease symptoms ↓ Fatality ↓ Parasitemia (rebounds 9 days after stopping treatment) ↓ Vascular leakage of blood brain barrier (BBB) ↓ Infiltrating lymphocytes	(29)
		Treated with Trametinib orally starting 6 h post-infection	Trametinib: ↓ Neurological Symptoms ↓ Fatality ↓ Parasitemia ↓ Vascular leakage of BBB ↓ Infiltrating lymphocytes	
**Trametinib**				
Female C57BL/6 mice	IAV	Viral Titers: Pre-treated with Trametinib twice daily starting 1 h prior to infection	↓ Viral Replication	(30)
		Body Weight and Symptoms: Treated with Trametinib once daily	↓ Weight Loss Delayed noticeable clinical symptoms	
Male C57BL/6 mice	*S. aureus* (femur, fracture injury model)	Hydrogel Construct: Vancomycin + Rifampicin + Trametinib (VRT) Controls included		(31)
		Model I (Local) Immediate hydrogel treatment within first 15 min of infection [to prevent osteomyelitis]	New cartilage formation in VRT groups 1 week post-infection	
		Model II (Systemic) Infection + antibiotics within 15 min of infection + antibiotics twice daily for 14 days. Once weekly VRT hydrogel injection into fracture site or oral trametinib.	↓ Serum G-CSF, IL-6, MCP-1, IP-10, MIP-1β, GM-CSF, IL-17, TNFα ↑ Fracture Healing Scores at 4 weeks post-infection	
Male C57BL/6 mice	Arthritis Model (intra-articular knee joint infection with *S. aureus*, heat-killed (HK) *S. aureus*, LPS, TiO_2_, and MSU)	Vancomycin and Rifampicin for 6 days post-MRSA infection Treated with Trametinib subcutaneous for 3 days starting on day 4 after infection	↓ Inflammation, Synovial hyperplasia, Cartilage Degradation, Osteoclasts	(32)
Male C57BL/6 mice	*E. coli* LPS (i.p.)	Pre-treated with Trametinib by i.p. 1 h before challenge	↓ Blood Urea Nitrogen (BUN) ↓ Renal Cortical KIM-1 transcript levels	(33)
Male C57BL/6 mice	*E. coli* LPS-ALI	Pre-treated with Trametinib orally for 3 days	↓ Lung Injury Score ↓ Edema, Protein in BALF ↓ IL-1β, MCP-1, TNFα, MPO activity, MDA levels	(34)
Mice	LPS + D-galactosamine (i.p.)	Trametinib oral dose	↓ Death at 12 h post-challenge ↓TNFα Resistant to endotoxin shock	(35)
Aged (40 week-old) Male C57BL/6 mice	CLP	Trametinib i.p. 6 h post-CLP	↓ TNFα, IL-1β, IL-6, GM-CSF (compared to CLP-only) No CLP-induced hypothermia Prevention of kidney injury	(36)
Female NMRI mice	Chronic *Schistosoma mansoni* (*S. mansoni*) infection	Treated with single oral dose of Trametinib	↓ Worm count	(37)
Female C57BL/6 mice	*L. amazonensis* (hind footpad)	Pre-treatment with Trametinib orally for 1 week prior to infection, continuing until endpoint.	↓ Lesion Size ↓ Parasites in lesion ↓ MIP-1α, MCP-1	(38)
		Treated with Trametinib by oral gavage starting 6 weeks post-infection continuing until endpoint.	↓ Lesion Size ↓ Parasite Burden	
Male C57BL/6 mice	*Salmonella typhimurium* (*S. Tm*) strain LT2 (oral gavage, enterocolitis model)	Pre-treatment with Streptomycin by oral gavage 24 h prior to infection with Trametinib by oral gavage 4 h post-infection	↓ Bacterial Burden in feces, small intestine, and spleen ↓ Splenomegaly Improvement of intestinal pathology	(39)
**PD0325901**				
C57BL/6 mice	*E. coli* LPS (oropharyngeal)	Treatment with PD0325901 (i.p.) on days 1 and 3 post-challenge	↓ BAL cells ↓ PMNs	(40)
C57BL/6 mice	*P. aeruginosa* (oropharyngeal)	Treatment with PD0325901 (i.p.) on days 2 and 3 post-infection	↓ BAL cells ↓ PMNs	(41)
Female BALB/c mice	*F. tularensis* Live Vaccine Strain (intranasal)	Pre-treated with PD0325901 by oral gavage 24 h prior to infection	↓ CFUs in Livers and Spleens of pre-treatment groups (n.s. difference between all groups in lungs)	(42)
		Treated with PD0325901 48 h post-infection	↓ Spleen levels of IFN-γ, MCP-1, IL-6, TNFα (pre-treated mice)	
Male C57BL/6 mice	Destabilization of the medial meniscus (DMM) injury	Treated with PD0325901	↓ Osteoclasts ↓ NLRP3 inflammasome expression ↑ Bone/tissue volume ratio	(43)
Male C57BL/6 mice	*S. aureus* (osteomyelitis in femurs)	Treated with PD0325901 (i.p.) daily starting on day 5 post-infection All mice treated with Gentamicin (20 mg/kg/day) (i.p.) starting day 1 post-infection	↓ Cortical Bone Loss ↓ Bacterial Burden in bone marrow ↑ Bone mineral density and bone volume/tissue ratio Improved bone morphology and structure	(44)
C57BL/6 mice *Cftr*^tm1kth^ F508del homozygous mice	*S. aureus* (intranasal)	Treated with PD0325901 (i.p.) immediately prior to infection	↓ Body Mass Loss ↓ Total cells in BAL (Neutrophils)	(45)
**CI-1040**				
C57BL/6 mice	IAV	Pre-treated with CI-1040 *per os* 8 h prior to infection and every 8 h post-infection	↓ Viral Titers	(46)
Female C3HeB/Fej mice	*L. amazonensis* or *L. major* (hind footpad)	CI-1040 by oral gavage twice daily starting at day 0 for a total of 7 days	*L. amazonensis*-infected: ↑ CD40 surface expression on CD11c^+^ DCs ↓ IL-12p40 producing cells No effect on above markers in *L. major*-infected mice	(47)
Male KKAγ mice	High and Low-Fat Diet	CI-1040 orally twice daily	Suppressed upregulation of MEK1/2 activity in High-fat diet mice ↓ Non-fasting blood glucose level Improved glucose tolerance, insulin sensitivity	(48)
Male C57BL/KsJ-*db/db* mice (leptin-receptor deficient mice)	Diabetic Mouse Model	CI-1040 orally twice daily for 15 days	↓ ERK1/2 activity in visceral fat tissue Improved glucose tolerance, insulin tolerance Ameliorates dysregulation of adipocytokine expression	
**ATR-002**				
Male NMRI mice (pharmacokinetics)	Influenza A Virus	CI-1040 or ATR-002 by oral gavage or i.v.	More ATR-002 in the plasma after i.v. and oral gavage	(49)
Female C57BL/6 mice (viral titer and survival studies)		ATR-002 or CI-1040 three times daily (i.v.)	CI-1040: ↓ Viral Titer ATR-002: ↓ Viral Titer	
		ATR-002 twice daily starting at 24, 48, or 72 h post-infection	↑ Survival when treatment started 24 h post-infection	
Female C57BL/6 mice	Influenza A Virus	ATR-002 by oral gavage twice daily starting 24 h post-infection through day 3 with endpoint at day 6	↓ Induction of Tregs	(50)
Male and Female C57BL/6 mice	*S. aureus* (intranasally)	PD0325901 or ATR-002 (i.p.) immediately prior to infection	ATR-002: ↓ Bacterial Burden Both inhibitors: ↓ CXCL1 levels ↓ Loss of body mass ↓ Total Protein in BALF, BAL cells (neutrophils) at 24 h p.i.	(51)

## BACTERIAL EUKARYOTIC-LIKE SERINE/THREONINE PROTEIN KINASES

Similar to eukaryotic organisms, gram-negative and gram-positive bacteria have highly conserved genes encoding eukaryotic-like serine threonine kinases (eSTK). These proteins regulate diverse physiological pathways and are often counterbalanced by protein phosphatases to direct cellular responses to a wide variety of stimuli; mechanisms of eSTKs have been previously extensively reviewed (52–54). In *S. aureus,* Stk1 (*PknB*) is one of the better characterized eSTKs, which is known to function with its cognate phosphatase, Stp1 (55, 56). For example, a recent study demonstrated that phosphoregulation of *S. aureus* proteins by Stk1 and Stp1 regulates metabolic quiescence and antibiotic tolerance and specifically indicates that inhibition of Stk1 can function to present prevent persister colony formation and enhance sensitivity to antibiotics (57). One small molecule, called Inh2-B1, was designed to inhibit *S. aureus* Stk1 by binding to the ATP catalytic domain. Treatment of *S. aureus* with Inh2-B1 was demonstrated to alter cell wall biosynthesis and sensitize *S. aureus* to cephalosporin antibiotics (58). The anti-bacterial effect of Inh2-B1 was lost in a *S. aureus* mutant strain lacking Stk1 while activity was retained in a *S. aureus* mutant strain that retained Stk1 but lacked the phosphatase Stp1 (58). While Inh2-B1 did not elicit toxicity to mammalian cells, whether this compound interacted with mammalian proteins to potentially modulate host responses was not reported (58).

Interestingly, several mammalian targeting inhibitor compounds have recently been proposed to interact with *S. aureus* Stk1 and, therefore, elicit direct anti-bacterial effects. The MEK1/2 inhibitor CI-1040 and a refined version of its active metabolite, referred to as ATR-002, had direct anti-bacterial effects against *S. aureus* (59). However, in contrast to the anti-bacterial effect of Inh2-B1, the anti-bacterial effect of ATR-002 was not lost in a *Stk1* mutant strain though it no longer had activity in a background when both *Stk1* and *Stp1* were lost (59). What accounts for these different results between Inh2-B1 and ATR-002 remains to be fully determined. However, results could be impacted by possible variabilities in the genetic backgrounds or growth conditions that alter the susceptibilities of *S. aureus* to these compounds. ATR-002 may also have other targets beyond *Stk1*, or that there are compensatory mechanisms that negate the effects of ATR-002 on a *Stk1* mutant background. Another example of a mammalian targeting compound that elicits anti-bacterial effects is Loratadine. This is an FDA-approved antihistamine that also inhibits biofilm formation in *S. aureus* and *S. epidermidis* and reduces disease severity in a *S. aureus* pulmonary infection murine model (60–62). The dual host-targeting and bacterial-targeting effects of these compounds provide new avenues of research to translate these potential beneficial properties to therapeutic clinical treatments.

## ANTI-VIRAL EFFECTS OF MEK1/2 INHIBITORS

It has now been just over 25 years since an initial discovery that MEK1/2 inhibitor compound U0126 disrupted influenza A virus replication during *in vitro* infection by inhibiting nuclear export of viral ribonucleoproteins (63), a finding also then reported for influenza B virus infection (64). Subsequent *in vivo* studies demonstrated that U0126 treatment of mice prior to influenza A virus infection decreased virus titers in the lung and increased survival (19, 20), findings that produced similar results using the MEK1/2 inhibitor compounds Trametinib (30), CI-1040 (46, 49), and ATR-002 (49). In additional *in vivo* experimental models, pre-treatment and daily treatment of BALB/c mice with the MEK1/2 inhibitor U0126 decreased viral titers in brain tissue following intracranial infection with yellow fever virus (17). In a rabies virus intracerebral infection of Swiss Albino Mice, treatment with U0126 on days 0, 2, 4, and 6 post-infection improved survival and decreased viral titers and decreased production of inflammatory cytokines (18). In addition, MEK1/2 inhibitors have also been recognized to interfere with replication of several other viruses, including SARS-CoV-2, respiratory syncytial virus (RSV) (65), and Dengue and Zika viruses (66). A recent review further details the anti-viral capabilities and mechanisms of MEK1/2 inhibitor compounds (67).

A key development in the field occurred when the MEK1/2 inhibitor ATR-002, which also interferes with influenza virus replication (49, 59, 68), was demonstrated to disrupt SARS-CoV-2 replication (69, 70). These findings provided the opportunity to test the antiviral effects ATR-002 in a human phase II clinical trial for treatment of hospitalized adults with moderate to severe SARS-CoV-2 infection, enrolling *n* = 51 patients in the placebo arm and *n* = 50 patients in the ATR-002-treated arm of the study; though overall enrollment targets for the study were not met, ATR-002 exhibited low toxicity with potential benefits including reduced pro-inflammatory mediators, such as IL-6, and reduced viral levels at some collection points during the study (16, 71). Given these results, potential application of the MEK1/2 inhibitor ATR-002 as a dual host and pathogen targeting compound for treatment of viral induced lung pneumonia is intriguing. Additional studies to uncover how the MEK1/2 pathway regulates epithelial cell responses in the presence and absence of respiratory infections would increase the understanding of the underlying biological antiviral mechanisms regulated by MEK1 and MEK2.

## HOST TARGETING EFFECTS OF MEK1/2 INHIBITORS DURING GRAM-NEGATIVE BACTERIAL INFECTIONS

Multiple experimental models have been used to investigate the therapeutic potential of MEK1/2 inhibitor compounds in the context of gram-negative bacterial infections, including the use of live bacterial infection and initiation of inflammatory injuries using sterile bacterial components, such as LPS. Delivery of LPS to the lung is a commonly used sterile model of acute lung injury (ALI), with peak ALI on days 1–2 and resolution of injury and inflammation observed by days 4–7 after injury (72, 73). While routinely used in experimental models, pre-treatment with compounds such as MEK1/2 inhibitors likely do not reflect human clinical scenarios though can serve as a useful initiating point for evaluating the therapeutic potential of a compound. Thus, multiple studies have employed pre-treatment with MEK1/2 inhibitors prior to initiation of an injury. For example, pre-treatment with the MEK1/2 inhibitor Trametinib decreased inflammatory responses in a mouse model of peritoneal *E. coli* LPS challenge (33) and pre-treatment also decreased lung injury following *E. coli* LPS-ALI (34). In a mouse model of endotoxin shock using peritoneal delivery of LPS and D-galactosamine, Trametinib prevented death while reducing the inflammatory response (35). Other experimental mouse models administering either U0126 or PD98059 MEK1/2 inhibitors 2 h prior to LPS challenge demonstrated decreased inflammatory responses (23), while in a rat model of *H. pylori* LPS-induced gastritis PD98059 treatment resulted in a modest decrease in the inflammatory response (28). Furthermore, the inflammatory response and injury from an LPS-ALI in mice previously infected with RSV could be reduced by treatment with PD98059 (74). While these prophylactic treatment studies establish the anti-inflammatory potential of MEK1/2 inhibitors on a sterile host response, these models differ in timing from when a human clinical therapeutic treatment would be provided. To better model a therapeutic treatment, a study providing PD0325901 treatment on days 1 and 3 after initiation of *E. coli* LPS-ALI in C57BL6/J mice reported decreased neutrophil recruitment to the alveolar space on day 4 after LPS-ALI, consistent with anti-inflammatory effects described in many of the prophylactic studies (40).

Sterile experimental models of inflammation and injury are commonly employed research models; however, procedures incorporating the use of infectious organisms are likely better models of human disease. One commonly used model of sepsis is the cecal ligation and puncture (CLP) model (75). The MEK1/2 inhibitor Trametinib delivered following CLP in C57BL/6 mice decreased inflammatory cytokines and prevented CLP-induced hypothermia while preventing kidney injury (36). Furthermore, a CLP model in Sprague Dawley Rats also demonstrated that pre-treatment with PD98059 reduced lung injury and MPO activity, consistent with a decreased neutrophilic inflammatory response (27). In a pulmonary infection model of C57BL6/J mice using oropharyngeal delivery of *Pseudomonas aeruginosa* strain K, treatment with the MEK1/2 inhibitor PD0325901 on days 2 and 3 after infection reduced neutrophil recruitment to the alveolar space on day 4 compared to vehicle-treated mice (41), demonstrating that MEK1/2 inhibitors have the potential to be delivered in a therapeutic window after initiation of an infection. While the *P. aeruginosa* eSTK PpkA has been well described in the literature (76, 77), direct effects of MEK1/2 inhibitors on *P. aeruginosa* have not been reported. Collectively, these studies demonstrate that MEK1/2 inhibitors can be utilized to decrease inflammatory responses to gram-negative bacteria or their products, such as LPS, during acute settings, though additional preclinical studies that combine experimental use of MEK1/2 inhibitors with standards of care such as antibiotic regimens could provide a more comprehensive assessment of how MEK1/2 inhibitors impact gram-negative bacterial pathogenesis.

Other experimental models of gram-negative intracellular bacterial pathogens have also evaluated the potential therapeutic effects of MEK1/2 inhibitors. *Salmonella* is a facultative intracellular pathogen that actively remodels intracellular compartments to establish the *Salmonella* containing vacuole (SCV) (78). During *Salmonella* Typhimurium (*S*. Tm) infection, the effector protein SopB was identified to mediate host cell Cdc42-dependent vimentin remodeling around the SCV and multiple MEK1/2 inhibitor compounds, including Trametinib, TAK-733, Pimasertinib, AZD8330, RDEA119, and BI-847325, were identified in a screen to disrupt this process (39). *S*. Tm growth in media containing the MEK1/2 inhibitor Trametinib was not reduced compared to media containing vehicle alone, suggesting that Trametinib, at 1 µM, did not have direct anti-bacterial effects (39). However, whether higher doses of Trametinib or if other MEK1/2 inhibitor compounds have direct anti-bacterial effects on *Salmonella* remains unresolved. Interestingly, previous work using interferon-gamma stimulated mouse RAW 264.7 macrophages demonstrated that treatment with either of the MEK1/2 inhibitors PD98059 or U0126 reduced *Salmonella* Tm filamentation in cells although this was associated a moderate impairment in intracellular bacterial killing (79). Whether there is a differential requirement for the MEK1/2 pathway in regulating intracellular *Salmonella* in different cell types has not been fully investigated, however, future studies investigating the effects of MEK1/2 inhibitors on *Salmonella* pathogenesis in primary human cells would be informative.

MEK1/2 inhibitors have also been studied in the context of the facultative intracellular gram-negative bacterial pathogen *Francisella tularensis*. After phagocytosis by macrophages, *F. tularensis* virulence factors disrupt macrophage phagosome maturation and escape to the macrophage cytosol to replicate (80). A study using the attenuated *F. tularensis* live vaccine strain (LVS) demonstrated that daily treatments with the MEK1/2 inhibitor PD0325901, starting either 24 h prior to intranasal infection with *F. tularensis* LVS or 48 h post-infection, resulted in decreased ERK1/2 phosphorylation in mouse lungs at 96 h after infection (42). At this endpoint, bacterial burdens in the liver and spleen were modestly reduced, as was spleen inflammatory cytokines, in mice that had received pre-treatment with PD035901 but not in the post-treatment group, while neither PD0325901-treatment group altered bacterial burden or the inflammatory response in the lung and did not improve survival (42). Whether MEK1/2 inhibitors impact *F. tularensis* escape from the macrophage phagosome, intramacrophage replication, or if MEK1/2 inhibitors have direct anti-bacterial effects on *Francisella* has not been reported. An additional study utilizing *Burkholderia pseudomallei* infection of the macrophage cell line MH-S and treated with PD0325901 demonstrated no alteration of intracellular bacterial replication, although PD0325901 could result in reduction in TNF secretion 4 hours after infection (81). This study also did not evaluate whether the MEK1/2 inhibitor had direct anti-bacterial effects on *Burkholderia*. Overall, the function of eSTKs in *Francisella* and *Burkholderia* has not been widely reported. While *F. tularensis* is recognized to have a type VI secretion system that is largely encoded by genes in the *Francisella* pathogenicity island (FPI) (82), none of the FPI genes were reported to have homology to the eSTK *Ppka* that has been described as part of the *P. aeruginosa* type VI secretion system (76, 83). In contrast, *B. pseudomallei* was reported to have an ortholog of PpkA (76).

Overall, the literature support beneficial host-targeting anti-inflammatory effects of MEK1/2 inhibitors during acute gram-negative infections or experimental models of endotoxin shock or sepsis. However, direct anti-bacterial effects of MEK1/2 inhibitor compounds on gram-negative pathogens have not been reported in contrast to the direct anti-bacterial effects on several gram-positive organisms that are major human pathogens. Whether MEK1/2 inhibitors cannot enter gram-negative organisms or are rapidly subjected to efflux mechanisms remains unknown, but additional investigation using mutant strains defective in efflux could provide insight into this area (84). Further studies to determine if MEK1/2 inhibitors impact gram-negative bacterial responses to environmental stresses, including antibiotics, could also uncover new directions for the field.

## DUAL HOST AND PATHOGEN TARGETING EFFECTS OF MEK1/2 INHIBITORS DURING GRAM-POSITIVE BACTERIAL INFECTION

Multiple MEK1/2 inhibitor compounds have also been used in the context of infections with gram-positive organisms. The MEK1/2 inhibitor PD0325901 was provided by intraperitoneal injection at the time of intranasal infection of mice with 1 × 10^7^ *S. aureus* USA300. This study demonstrated a beneficial anti-inflammatory effect of PD0325901 treatment compared to vehicle treatment in C57BL6/J mice and in the cystic fibrosis *Cftr^tm1kth^* mouse model (45). Specifically, C57BL6/J mice treated with PD0325901 had decreased infection-induced loss of body mass and had significantly less inflammatory cell recruitment to the alveolar space, which was predominately a reduction of neutrophils, without impacting bacterial CFU compared to vehicle-treated mice. This study did not identify an *in vivo* anti-bacterial effect of PD0325901 and did not investigate if potential *in vitro* anti-bacterial effects of PD0325901 were comparable to the effects described for CI-1040 and ATR-002 (45). However, a subsequent study directly compared the *in vitro* anti-bacterial effects of the MEK1/2 inhibitor compounds CI-1040, PD0325901, Trametinib, and ATR-002, demonstrating that ATR-002 had a unique ability to reduce growth of *S. aureus* USA300 in broth culture (51). The anti-bacterial effect of ATR-002 was also confirmed on 40 clinical isolates of *S. aureus* obtained from people with cystic fibrosis (51). Furthermore, an *in vivo* intranasal infection model in C57BL6/J mice demonstrated that while both MEK1/2 inhibitors ATR-002 and PD0325901 had comparable abilities to reduce disease and decrease the inflammatory response, only ATR-002 also significantly reduced *S. aureus* CFU in the lung when quantified 4 h after infection (51). Overall, this study demonstrated that ATR-002 retained its anti-bacterial effect on clinically relevant strains and that ATR-002 could elicit an anti-bacterial effect *in vivo*.

MEK1/2 inhibitors have also been used in other non-pulmonary murine models of *S. aureus* infection. For example, a femur fracture model with MRSA infection tested the MEK1/2 inhibitor Trametinib as an adjuvant to antibiotic treatment and noted beneficial fracture healing and a decreased inflammatory response in conditions containing Trametinib (31). The antibiotic regimen used in this study effectively reduced bacterial CFUs and potential anti-bacterial effects of Trametinib single treatment were not investigated (31). In related findings, Trametinib addition to antibiotic treatment reduced inflammation in a mouse model of intraarticular knee joint infection with MRSA though potential direct anti-bacterial effects of Trametinib were not reported (32). In a murine model of *S. aureus* osteomyelitis, the MEK1/2 inhibitor PD0325901 or vehicle control was each provided in addition to gentamicin treatment, and interestingly, mice treated with PD0325901 had decreased disease severity but also had decreased bacterial burdens (44). Additional insights into how MEK1/2 inhibitors, especially ATR-002, may influence the host response to *S. aureus,* including biofilms or small colony variants, and whether direct anti-bacterial effects of ATR-002 can synergize with host antimicrobial responses to enhance killing and clearance of *S. aureus* should be further investigated.

While literature on the potential antimicrobial effects of MEK1/2 inhibitor compounds on *S. aureus* infection models is growing, it was also previously reported, though data not shown, that the MEK1/2 inhibitor ATR-002 did not affect *in vitro* growth of *Mycobacterium abscessus*, but did affect growth of *Bacillus subtilis* and *Streptococcus pneumoniae* (59). In addition, one study that infected human MDSCs with *M. tuberculosis* demonstrated that treatment with the MEK1/2 inhibitor PD98059 after infection reduced CFUs over a 48-h culture period. However, whether these results were sustained for longer incubation times that likely better capture *M. tuberculosis* growth was not evaluated (85). Whether MEK1/2 inhibitor compounds exert anti-bacterial effects on different *Mycobacteria* species, either directly on the bacteria, or indirectly through manipulation of the host cell, remains to be fully determined. Overall, additional studies evaluating the potential dual host and pathogen targeting of MEK1/2 inhibitor compounds during acute and chronic infections with gram-positive bacteria should be further explored, with experiments designed to provide evidence needed to bridge the gap to human clinical trials.

## MEK1/2 INHIBITORS TREATMENT DURING PARASITIC INFECTIONS

The experimental use of MEK1/2 inhibitor compounds has also been reported in models using other pathogenic microbes. For example, amastigote *Leishmania amazonesis* infection of mouse bone marrow-derived macrophages induces ERK1/2 activation-dependent IL-10 production that can be reduced by adding either of the MEK1/2 inhibitors U0126 or PD98059 (22). MEK1/2 inhibitor addition during this infection reduced histone H3 phosphorylation and reduced recruitment of Sp1 to the *Il10* promoter (22). After establishment of amastigote *L. amazonensis* footpad infection in BALB/c mice for 18 days, weekly treatment with U0126 compared to vehicle control resulted in a significant reduction in lesion size and parasite burden, consistent with the ability of MEK1/2 inhibitors to prevent IgG-mediated exacerbation of disease (22). A more recent study using RAW 264.7 macrophages demonstrated that treatment with the MEK1/2 inhibitor Trametinib decreased macrophage phagocytosis of *L. amazonensis* amastagotes but did not alter intracellular growth over a 5-day period (38), while an additional report demonstrated that U0126 decreased mouse macrophage phagocytosis of *L. amazonensis* promastigotes (21). Separately, it was reported that the MEK1/2 inhibitor PD98059 or CI-1040 alter macrophage and dendritic cell activation in *in vitro* and *in vivo,* respectively, though whether this is related to phagocytosis or replication *L. amazonensis* is unknown (47). Two different *in vivo* mouse footpad infection models also demonstrated beneficial effects of MEK1/2 inhibitor treatment during infection. First, pre-treatment of mice prior to infection with Trametinib decreased lesion size and reduced parasite burden (38). Second, initiation of Trametinib treatment at 6 weeks after infection also attenuated lesion size and reduce parasite burden (38). However, in contrast to these findings, a study that infected the footpad of female BALB/c mice with *L. amazonensis* and provided daily U0126 treatment for 7 days starting at 4 weeks after infection reported increased lesion size in the U0126-treated group and an increase in parasite burden 1 week following U0126 treatment (21). While it is not clear how these conflicting reports can be fully explained, the timing, dose, and length of treatment following infection, hormonal differences at the time of infection or treatment, or differences in the microbiome between animals may be contributing factors that should be investigated in a more comprehensive manner in future studies.

In additional to these *Leishmania* studies, several other areas of parasitology have also evaluated the use of MEK1/2 inhibitor compounds. For example, one study screened 114 compounds from the National Cancer Institute’s Developmental Therapeutic Program against *Schistosoma mansoni* larvae and adult worms (37). One top hit found in this screen was the MEK1/2 inhibitor Trametinib, and a single dose of Trametinib administered to mice harboring chronic *S. mansoni* infection resulted in decreased worm burden. These direct anti-parasitic effects were also observed in adult worm pairs treated with U0126, resulting in worm uncoupling and suppressed egg release (86). Using a rodent malaria parasite, *Plasmodium berghei* ANKA (PbA), infection model of experimental cerebral malaria in mice, administration of either MEK1/2 inhibitors PD98059 or Trametinib starting 6 h after infection and provided daily for 7 days resulted in decreased disease and reduced parasitemia that enhanced overall survival over an approximately 4-week period (29). While parasitemia was reduced during the *in vivo* treatments, *in vitro* studies using *P. falciperium* did not indicate direct anti-parasitic effects of PD98059 when analyzed compared to untreated or chloroquine-treated controls, and PbA infected blood treated *ex vivo* with PD98059 did not appear to restrict parasite growth (29). However, other studies have reported that Trametinib treatment interferes with *P. falciparum* and *P. knowlesi* proliferation in erythrocytes though it did not have an effect on the canine pathogen *B. divergens* (87). Interestingly, the MEK1/2 pathway was also previously identified to be important during erythrocyte infection (88, 89), with the MEK1/2 inhibitor compounds PD98059, U0126, and CI-1040 inhibiting *P. falciparum* proliferation in erythrocytes while U0126 and CI-1040 reduced *P. berghei* proliferation in erythrocytes, and U0126 was reported to reduce *P. berghei* proliferation in HepG2 cells (89). What accounts for discrepancies in these reports for different abilities to inhibit replication of parasites in erythrocytes is not clear. However, in the *in vivo* experimental cerebral malaria model, Trametinib greatly reduced brain inflammation and was found to increase IgM secretion from B-cells and to result in increased phagocytosis of parasites by macrophages and neutrophils (29). Overall, these results suggest there are multiple potential important host-targeting effects of MEK1/2 inhibitors, including disruption of parasite replication in erythrocytes and modulation of immune cell responses, though further clarity on specific mechanisms is needed. Whether some MEK1/2 inhibitor compounds have additional direct anti-parasite effects could be further investigated.

## INTERACTION OF MEK1/2 INHIBITORS WITH NEUTROPHILS

Tissue damage due to deleterious inflammatory responses underlies pathogenesis of many infectious diseases, including acute bacterial or viral pneumonia as well as chronic infections that persist in people with cystic fibrosis (90, 91). While numerous *in vivo* uses of different MEK1/2 inhibitor compounds demonstrate anti-inflammatory effects that include reductions in neutrophil numbers following infection or injury, distinguishing direct effects of MEK1/2 inhibitors on neutrophils compared to indirect effects such as reducing neutrophil recruitment by dampening of chemokines could use further exploration. However, multiple *in vitro* studies have highlighted potential roles of MEK1/2 in regulating potential neutrophil functions. Consistent with reduced neutrophil recruitment, *in vitro* neutrophil migration in response to LPS or the TLR2/1 stimuli Pam3CSK4 was reduced by treatment with the MEK1/2 inhibitor U0126 (92).

Other important bactericidal functions of neutrophils requires critical regulation of NADPH oxidase activity (93), and multiple studies have identified regulatory roles of the MEK1/2 pathway. For example, a study that preincubated human neutrophils with 50 µM PD98059 resulted in significant of inhibition MEK1/2 activation and NADPH oxidase activity following stimulation with fMLP or opsonized zymosan (94). Other work demonstrated a similar concentration of PD98059 reduced phosphorylation of the p67^phox^ subunit of the neutrophil NADPH oxidase (95). While subsequent studies suggest that these concentrations of PD98059 may have additional off-target effects beyond MEK1/2 (96, 97) other studies have also identified roles of MEK1/2 in regulating neutrophil NADPH oxidase function. For example, a study using mouse neutrophils demonstrated that MEK1/2-ERK1/2 activation downstream of EphA2 receptor mediated recognition of opsonized *C. albicans* yeast was involved in priming of the p47^phox^ NADPH oxidase subunit through phosphorylation at Ser345 (98). This result is consistent with the previously recognized priming of p47^phox^ Ser345 by the MEK1/2-ERK1/2 pathway following stimulation with GM-CSF, while the p38 pathway mediated TNF-dependent phosphorylation at Ser345 (99). Overall, there is good evidence that the MEK1/2 pathway has a role regulating neutrophil NADPH oxidase function though this role may differ based on stimuli or phagocytic cargo.

The production of neutrophil extracellular traps (NETs) has also been linked to MEK1/2 pathway activation through activation of the NADPH oxidase based on studies that used the MEK1/2 inhibitor U0126 (100). This study noted that U0126 did not affect neutrophil phagocytosis or degranulation, and in other studies of human neutrophils, treatment with MEK1/2 inhibitors PD0325901, Trametinib, or CI-1040 did not impar phagocytosis or phagosome acidification of opsonized *E. coli* or *S. aureus* pHrodo bioparticles (45). Furthermore, pre-treatment of neutrophils with PD0325901 for 2 h prior to infection with live *S. aureus* was reported to increase bacterial killing (101). As there is no indication of PD0325901 having direct anti-bacterial effects on *S. aureus in vitro* (51), modulation of neutrophil responses is likely indicated for the enhanced bacterial killing although no specific mechanisms were reported or proposed (101). Collectively, additional evaluation of how different MEK1/2 inhibitor compounds alter human neutrophil interactions with pathogenic bacteria may yield further insight into how MEK1/2 inhibitors can modulate disease pathogenesis. With the unique anti-bacterial properties of ATR-002, dose response studies to evaluate the *in vitro* effects of this compound on human neutrophil antimicrobial properties should be performed with additional *in vivo* investigations to uncover how ATR-002 may affect the host defense response during acute and chronic infections. Furthermore, additional insights into whether MEK1 and MEK2 have differential roles in regulating neutrophil functions remains to be fully determined.

## INTERACTION OF MEK1/2 INHIBITORS WITH MACROPHAGES

Multiple studies have investigated the role of the MEK1/2 pathway in regulating macrophage functions by applying MEK1/2 inhibitor compounds during experimental assays. Similar to findings with human neutrophils, the MEK1/2 inhibitor compounds PD0325901, Trametinib, or CI-1040 did not alter phagocytosis or phagosome acidification of opsonized *S. aureus*, *E. coli*, or zymosan pHrodo bioparticles but did decrease inflammatory cytokine production following stimulation with LPS, Pam3CSK4, or FSL1 (45). The MEK1/2 inhibitor ATR-002 also did not alter human monocyte-derived macrophage phagocytosis or phagosome acidification of opsonized *S. aureus* pHrodo bioparticles (51). However, whether ATR-002 or any other MEK1/2 inhibitors modulate phagocytosis and killing of live *S. aureus* has not been determined. In addition, as intramacrophage *S. aureus* survival has been described (102, 103), whether ATR-002 can interact with *S. aureus* in these intracellular locations is unknown. Interestingly, it was reported that EGFR-MEK1/2 pathway activation in murine macrophages decreased mitochondrial reactive oxygen species production and resulted in suppressed macrophage bactericidal capabilities; a phenotype that could be reversed by the addition of the MEK1/2 inhibitor PD0325901 (44). Whether this pathway had a similar function in human macrophages has not been reported.

While the regulation of TLR2 and TLR4-dependent inflammatory responses can be decreased by the addition of MEK1/2 inhibitors to macrophages, MEK1/2 inhibitors have also been reported to modify the response of macrophages to other TLRs and cytokines. For example, mouse macrophage treatment with the MEK1/2 inhibitor PD0325901 increased IL-4/IL-13 induced gene and protein expression *in vitro* and *in vivo* following lung injury (40) or infection (41). Furthermore, treatment with the MEK1/2 inhibitor PD0325901 alone was sufficient to increase the efferocytosis abilities of human and mouse macrophages, likely through increasing the expression of macrophage efferocytosis receptors (40). In addition, MEK1/2 inhibitors increase macrophage reverse cholesterol transport and reduce inflammation in atherosclerosis models (104, 105), phenotypes that may also be linked to macrophage programming and efferocytosis. MEK1/2 inhibitors have also been reported to increase macrophage IRF1-dependent interferon programming following TLR7 stimulation (106). Collectively, MEK1/2 inhibitors appear to have broad abilities to modulate macrophage inflammatory responses, and further investigation into these responses should be performed in the context of current approved cancer therapies and in future potential uses during infections disease.

## DIFFERENTIAL ROLES OF MEK1 AND MEK2 IN REGULATING THE INFLAMMATORY RESPONSE

More recent studies highlight potential distinct roles of MEK1 and MEK2 that have been uncovered through experimental designs not dependent on MEK1/2 inhibitor compounds that target both proteins. Mouse models have demonstrated that full-body knockout of *Mek1* is embryonic lethal, due to defects in placental development (107, 108), while in contrast, mice with a full-body knockout of *Mek2* are viable (109). Conditional knockout of *Mek1* using the *Lyz2* Cre-flox system retained viability in mice while achieving broad MEK1 knockout in myeloid cells, and *E. coli* LPS-ALI resulted in a significantly increased and prolonged pro-inflammatory response in mice lacking myeloid MEK1 compared to controls (110). In this model, mice lacking myeloid MEK1 had sustained or increased phosphorylation of ERK1/2 and phosphorylation of MEK2 in macrophages, monocytes, and neutrophils after LPS-ALI, suggesting that unrestrained MEK2 activation prolonged detrimental inflammatory responses (110). This phenotype is likely consistent with other work demonstrating a critical regulatory role of MEK1 to decrease MEK1/2-ERK1/2 pathway activation in a negative feedback loop (4). In support of a model of MEK2 driving detrimental inflammatory responses, a SNP in *MAP2K2* was discovered to be associated with worse outcomes in human ARDS although a functional effect on MEK1/2 signaling of these SNPs have not been described (111). However, *Mek2^−/−^* mice subjected to either *E. coli* LPS-ALI or pulmonary infection with *Pseudomonas aeruginosa* strain K demonstrated that loss of MEK2 was protective during ALI compared to wild-type, having decreased injury and neutrophil recruitment (111). In addition, *Mek2^−/−^* mice were also protected following intranasal infection with MRSA strain USA300 (51). Whether myeloid cell conditional deletion of *Mek1* also impaired the resolution of ALI following gram-positive bacterial infection remains unknown.

## ALTERNATIVE COMPOUNDS WITH DUAL HOST PATHOGEN TARGETING

There are likely many other examples of compounds that elicit both host and pathogen targeting effects. For example, the macrolide antibiotic azithromycin has been recognized to impact the inflammatory response that is, at least in part, due to the modulation of macrophage programming (112, 113). On the other hand, repurposing mammalian targeting compounds as potential anti-bacterial agents is also a very active area of research. One recent study screened 38 host-targeting kinase inhibitors (although none of the 38 inhibitors were MEK1/2 inhibitors) and discovered that Sorafenib, an FDA-approved inhibitor that targets VEGFR, PDGFR, and Raf kinases, demonstrated anti-bacterial activity against *Streptococcus pneumoniae* (114). This study provided *in vitro* anti-bacterial evidence of Sorafenib using laboratory and clinical isolates, and in an ALI infection model, Sorafenib treatment provided 1 h after infection and every 24 h provided a modest but significant survival benefit compared to placebo control, with 25% survival in the Sorafenib-treated group at day 4 with 0% survival in the placebo-treated group at day 3 (114). Identification of Sorafenib-derived compounds with activity against *S. aureus* was also previously reported although the *S. aureus* protein targeted by Sorafenib was not identified (115, 116). Experimental procedures to further screen mammalian targeting compounds for potential anti-bacterial effects against multiple pathogenic organisms may identify additional promising compounds that may have beneficial dual host and pathogen targeting properties; including MEK1/2 inhibitors such as ATR-002 as controls in these studies would provide useful comparative results.

## FUTURE DIRECTIONS

Development of MEK1/2 inhibitor compounds has provided multiple tools for laboratory research to investigate the roles of MEK1/2 in regulating inflammatory and immune responses during many different models of human disease. In the context of translational use in human infections, one concern of therapeutic anti-inflammatory treatments is that the treatment will result in impaired host defense mechanisms and increase the pathogenesis of infection. One cautionary example is the halted trial for use of LTB4 receptor antagonists in people with cystic fibrosis due to increased adverse pulmonary events (117). Further experimental testing revealed that reduced neutrophil recruitment was associated with impaired clearance of *P. aeruginosa* in an experimental mouse model (118). Identification of dual host and pathogen targeting therapeutic compounds may help circumvent similar pitfalls in human clinical trials.

While the results reviewed here provide a positive outlook for the dual host and pathogen targeting capabilities of MEK1/2 inhibitor compounds, one limitation of preclinical experimental testing of therapeutics is the potential for bias toward reporting of positive results in the literature. An additional confounder we noticed in the literature is the tendency of studies to misreport the use of MEK1/2 targeting inhibitor compounds as ERK1/2 inhibitors; while decreased ERK1/2 phosphorylation is observed during the use of MEK1/2 inhibitors, the compounds themselves are targeting MEK1 and MEK2, proteins that function upstream and may have ERK1/2 independent functions. While specific ERK1/2 targeting inhibitor compounds do exist, whether any of these compounds exert direct anti-bacterial effects is unknown. Other pitfalls of MEK1/2 inhibitors have been reported, including severe gastrointestinal toxicity (119) and a case report of pneumonitis after use of a MEK1/2 inhibitor in cancer therapy (120). Awareness of these complications should inform future clinical trials and screening for additional toxicity should be performed in preclinical *in vivo* experimental models. Many studies have not included sex as a potential biological variable that may impact experimental results and should be incorporated into future experimental design. Finally, experimental models that combine both human cells and small animal infection models should attempt to deliver therapeutic compounds after infection is established to better mimic clinical scenarios, and when possible, therapeutic compounds should be evaluated in models that better mimic complex co-infection or polymicrobial infections that include viral and secondary bacterial pneumonia as well as chronic infection models.
